# Vestibular Stimulation Causes Contraction of Subjective Time

**DOI:** 10.3389/fnint.2022.831059

**Published:** 2022-05-16

**Authors:** Nariman Utegaliyev, Christoph von Castell, Heiko Hecht

**Affiliations:** Psychologisches Institut, Johannes Gutenberg-Universität Mainz, Mainz, Germany

**Keywords:** time perception, subjective duration, vestibular system, postural load, balancing, temporal production vestibular system and time perception

## Abstract

As the cerebellum is involved in vestibular and time-keeping processes, we asked if the latter are related. We conducted three experiments to investigate the effects of vestibular stimulation on temporal processing of supra-second durations. In Experiment 1, subjects had to perform temporal productions of 10- and 15-s intervals either standing on both feet or while being engaged in the difficult balancing task of standing on one foot with their eyes closed (or open for control purposes). In Experiment 2, participants were required to produce intervals of 5, 10, 15, and 20 s while standing on both feet with their eyes open or closed, which constituted an easier balancing task. In Experiment 3, we removed the active balancing; temporal productions of the same four durations had to be performed with the eyes open or closed during the passive vestibular stimulation induced by the oscillatory movements of a swing. Participants produced longer intervals when their eyes were closed, but active balancing was not the culprit. On the contrary, temporal over-production was particularly pronounced during the passive vestibular stimulation brought about by the swing movements. Taken together, the experiments demonstrate that the contraction of the subjective time during balancing tasks with closed eyes is most likely of vestibular origin.

## Introduction

Time perception is one of the integral components of human consciousness and experience. Being able to time events, judge their durations, and establish their temporal order is of central importance to adaptive behavior, outcome judgment, and effective decision-making. However, the subjective duration of an event, in addition to its actual physical duration, can be affected by a variety of external factors, such as emotional content of the stimulus (Grommet et al., 2011), intensity of the sensory signal (Wearden et al., 2007), and attentional allocation (Brown, 1997). The present study focuses on the role of vestibular stimulation in time perception, which has thus far received little attention.

A successful model in the field of time perception, at least during prospective timing, where subjects are informed in advance that the duration of the stimulus or the event should be judged, is the Scalar Expectancy Theory (SET). According to this model, there is a hypothetical internal clock consisting of a pacemaker, an accumulator, and a switch (Treisman, 1963; Gibbon et al., 1984). The pacemaker emits pulses at a certain rate, the accumulator encodes the emitted pulses, and the switch connects the pacemaker and the accumulator. When a duration is to be produced or estimated, the switch closes, which allows the pulses from the pacemaker to be collected by the accumulator. When a to-be-timed interval is over, the switch opens, thus cutting the connection between the pacemaker and the accumulator. The number of pulses collected by the accumulator serves as an estimate of how much time has elapsed during the interval. Finally, the collected pulses representing subjective time are compared against duration representations stored in the long-term memory, and the duration judgment is made (Matthews and Meck, 2016). The more pulses collected by the accumulator during a given actual time period, the longer the subjective duration of the to-be-timed interval is perceived to be (Wearden, 2005). Even though the neurobiological basis for such an internal clock has remained elusive, SET continues to be an effective theoretical model for the explanation of various phenomena of subjective temporal experience (Buhusi and Meck, 2005).

Physiologically and/or emotionally arousing events have been demonstrated to reliably accelerate the rate at which the pacemaker emits pulses, thus leading to the lengthening of subjective time. Stimuli containing more intense perceptual stimulation are judged to be longer in duration. For instance, filled auditory intervals are perceived to last longer than empty intervals (Thomas and Brown, 1974; Wearden et al., 2007). Likewise, auditory and visual stimuli preceded by trains of clicks (Penton-Voak et al., 1996) and flickering visual stimuli (Kanai et al., 2006) were overestimated in duration compared to their counterparts with less sensory intensity. Regarding emotional content, it has been shown that people overestimated the durations of faces depicting intense emotional expressions such as anger or happiness (Droit-Volet et al., 2004) and pictures evoking fear (Grommet et al., 2011). A similar effect was found with emotional auditory stimuli; people overestimated the duration of negative sounds compared to positive ones (Noulhiane et al., 2007). Additionally, more direct manipulations of physiological arousals, such as the administration of dopaminergic agents (Lake and Meck, 2013) or increasing the body temperature (Wearden and Penton-Voak, 1995), have led to the elongation of subjective time. Within the SET framework, the standard explanation of these effects is that the state of higher arousal increases the number of pulses generated by the pacemaker during a given physical unit of time, causing subjective temporal dilation.

The allocation of attentional resources between a timing task and a non-temporal secondary task also influences perceived subjective duration, which has been demonstrated in a number of experiments, where subjects had to perform a timing task and a concurrent non-timing secondary task. The more cognitively demanding and resource-intensive the task to be performed simultaneously with the temporal task, the more variable and shorter time estimations were (Thomas and Weaver, 1975; Zakay and Block, 1996). For example, with increasing levels of workload and complexity, produced time intervals became shorter and less accurate when subjects had to reproduce the duration of a text passage to which they were listening (Brown and Boltz, 2002). Likewise, being engaged in cognitive and motor tasks with increasing levels of difficulty leads to shorter estimates of perceived time in prospective paradigms (Brown, 1985, 1997; Zakay, 1998). The reasoning behind these findings is that as less attention is dedicated to the timing task, fewer pulses are encoded by the accumulator, which in turn results in shorter perceived durations. Note that also task-irrelevant information in the timing task itself can reduce accumulator performance (Thönes et al., 2018). Conversely, as more resources are allocated to temporal processing, the more pulses reach the accumulator, thus leading to temporal expansion.

What has come to play a prominent role in time perception research are theories of embodied cognition (Wittmann, 2014). Embodied cognition assumes that mental representations are situated in or referenced with respect to the body of the perceiver, and thus bodily changes of the latter should affect these representations (Leitan and Chaffey, 2014). For instance, still photographs depicting people, animals, and abstract images that are suggestive of dynamic motion were judged to be longer in duration compared to images with a standing posture, suggestive of a stationary body (Yamamoto and Miura, 2012). Similar effects of temporal overestimation were found with moving geometric forms, compared to stationary forms (Brown, 1995), with images of dancing sculptures featuring different intensities of implied dynamic motion (Nather and Bueno, 2011), and with animated drawings of a human walking at different speeds (Karşılar et al., 2018). This implies that the manipulation of visible body postures accelerates the rate of the pacemaker, which in turn produces more frequent pulses, thus dilating temporal experience.

In contrast to these examples of implied body motion, the investigation of the equivalent direct effects of actual bodily experience of the observer has received only limited attention in the time perception literature. A notable exception is a study where subjects had to wear a weighted backpack while performing a timing task of a visual stimulus. Perceived time was lengthened compared to the no-backpack condition, but note that this effect was observed when the to-be-timed stimulus was that of a backpack (Jia et al., 2015). The effect of the self-referential bodily experience on time perception was also demonstrated when subjects experienced an extended bodily discomfort induced by the submersion of the hand into cold water (Rey et al., 2017). Directing attention to oneself and/or the unpleasant bodily experience lengthened subjective time.

Considering the influence of physiological, attentional, and proprioceptive factors on subjective time, it is likely that the vestibular system likewise affects the pacemaker or the accumulator. In fact, the cerebellum, the subcortical brain structure, which is primarily responsible for motor coordination and vestibular control of balance, has been implicated in timing functions. Patients with damage to the cerebellum were more variable and less accurate in the production of rhythmic finger tapping and had poorer performance in duration discrimination tasks (Ivry and Keele, 1989). Similarly, Nichelli et al. (1996) reported impaired temporal discrimination for both sub-second and supra-second durations in patients who suffered from cerebellar degeneration. Consistent with these findings, repetitive trans-cranial stimulation over the left lateral cerebellum resulted in the overproduction of intervals in the sub-second range (Koch et al., 2007). When it comes to longer time intervals, however, the picture is less clear. Koch et al. (2007) failed to find similar effects for intervals in the supra-second range, whereas another study on patients with cerebellar lesions revealed that damage in the middle and superior cerebellum can lead to overproduction and underestimation of temporal intervals in this range, suggesting that impaired cerebellar activity slows down the pacemaker (Gooch et al., 2010). However, cerebellar involvement in the processing of supra-second durations awaits replication. Additionally, brain imaging studies suggested cerebellar activation during temporal processing tasks. Left lateral cerebellar activation was found to be prominent in fMRI studies, when participants were engaged in the temporal discrimination tasks of sub-second durations (Schubotz et al., 2000; Lewis and Miall, 2003).

Since the cerebellum is involved in the processing of vestibular and proprioceptive afferences (Rochefort et al., 2013), it seems plausible that vestibular activation or load would affect the mechanisms of time-keeping. Indeed, vestibular stimulation has been shown to affect temporal performance in a number of studies. One of the earlier studies on this topic exposed subjects to gravitational stress. They were seated in a cabin at the end of a centrifuge arm, pivoting in such a way that the force along the body’s g_z_-axis could be increased to 3 g. They had to reproduce temporal intervals of auditory tones of durations between 1 and 20 s by pressing and releasing a button. The reproduced intervals fell short of the stimulus durations in the 1 g control condition, and even more so under the gravitational stress induced by centrifugation (Frankenhaeuser, 1960). The author attributes the effect to reduced memory retention during centrifugation. Note that SET cannot easily explain this result. Changes in the pacemaker or accumulator should cancel out in reproduction tasks since perception and production should be equally affected. It appears that g-loading made subjects more impatient, if not forgetful, across the board.

A less complicated effect was found during otolith unloading, as tested in microgravity on three astronauts during a spaceflight mission. They first had to tap in synchrony with a metronome at inter-tap-intervals between 350 and 530 ms, which they successfully did. Then the metronome was turned off while they continued to tap. In microgravity the taps were faster than on the ground as if the pacemaker had sped up; additionally, it also led to increased variability of the inter-tap intervals (Semjen et al., 1998). More recently, the effect of vestibular stimulation on time perception was addressed by Capellia and colleagues (Capelli and Israël, 2007; Capelli et al., 2007). In one of their experiments, subjects were instructed to produce 1-s intervals by tapping a button before, during, and directly after being rotated by a mobile robot. The rotation could be at a constant angular velocity, at accelerating, or at decelerating rotation rates, and rotation would stop altogether between rotation phases. Inter-press intervals were not affected by the rotation *per se* or by the different velocity profiles; however, the intervals produced were more variable in all rotation conditions. The same observation was reported in another experiment using the same task of pushing a button each second, during the linear movement. Despite the clear effect of the vestibular stimulation on the accuracy of timed motor production, no systematic bias of pacemaker or accumulator was found in these studies. Furthermore, the increase in variability can not only be attributed to vestibular otolith stimulation but also to stress or changes in memory or motor response execution induced by the rotation, which occurred at maximally 60°/s.

Experiments where vestibular stimulation was induced by asking subjects to assume different bodily postures brought contradictory results as well. In a recent study conducted by Lo et al. (2021), subjects were instructed to produce durations with button presses of 3, 5, and 7 s while adopting body postures that signaled different levels of action (e.g., standing still, running). The temporal productions were shorter when assuming postures that signal action, which suggests that the dynamic posture has sped up the pulse rate of the pacemaker. In a study conducted by Schreuder et al. (2014), subjects had to produce considerably longer temporal intervals of 1.33, 1.58, and 2.17 min while assuming an upright or a supine posture and while at the same time being exposed to different odors: rosemary, peppermint, and no odor. Subjects exposed to rosemary odor under produced durations compared to peppermint and no odor condition. However, no effect of body posture was found, although it effectively had induced arousal measured by skin conductance response and heart rate. Thus, the potentially arousing effects of rosemary cannot account for its ability to speed up the clock.

**Figure 1:**
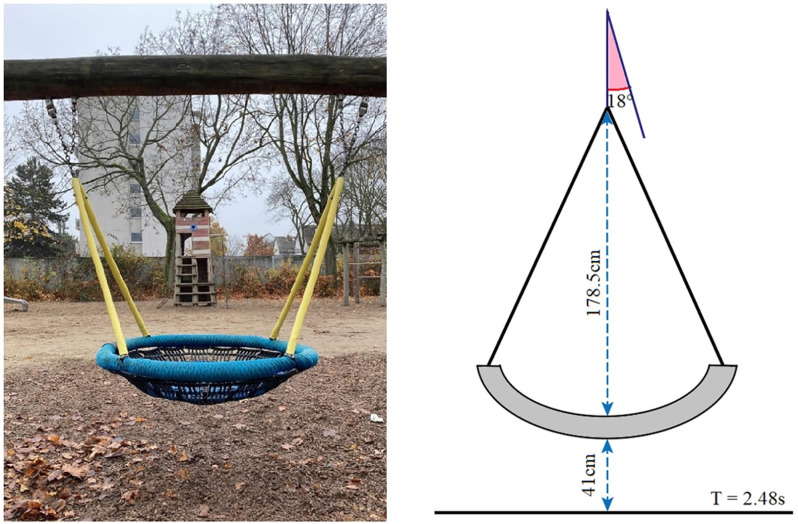
Nest swing used in Experiment 3.

As research on the influence of vestibular stimulation on time perception in supra-second durations is limited and has not always found a clear effect, other than increasing variability with vestibular excitation, we sought to take a closer look at supra-second time estimates in the face of vestibular engagement. Rather than stressing the vestibular system, we decided to add common balancing tasks to a temporal production task. Additionally, to minimize memory effects, we asked our subjects to produce a time interval of a given length rather than reproducing a previously perceived interval. The vestibular engagement was brought about by a difficult active balancing task (Experiment 1), an easier balancing task (Experiment 2), and a passive oscillatory movement induced by a swing (Experiment 3), which provided continuous vestibular acceleration stimulation without active balance control. In Experiment 1, subjects had to perform temporal production tasks of either 10 or 15 s while balancing on one foot with eyes closed or open. One-foot balancing is a challenging task. It requires the integration of information from vestibular and somatosensory sources to identify the position of the body in the environment prior to the execution of appropriate motor responses (Cherng et al., 2001). The absence of the visual information ordinarily used for fine-tuning makes this task rather challenging. Thus, the vestibular and proprioceptive cues necessary for balancing were either supplemented with visual information or not. In Experiment 2, temporal productions of 5, 10, 15, and 20 s were performed with eyes open or closed while standing on both feet. This is a much easier balancing task, which nevertheless introduces postural sway requiring an active balance maintenance (Era et al., 2006). It should significantly reduce the vestibular load and thus allow for an assessment of this information when comparing the results to those of Experiment 1. Finally, in Experiment 3, in contrast to the experiments above, the vestibular stimulation was induced passively. Subjects performed temporal productions of the same durations as in Experiment 2 while comfortably lying on a nest swing (see Figure 1) either when it was brought into an oscillatory motion or when it was at rest. Thus, it removed active postural control altogether. Within the SET framework, if the rate of the pacemaker is excited by the vestibular stimulation alone, we expect relative underproductions of temporal intervals during the balancing task. We also expect relative underproductions when subjects are in swinging motion compared to the stationary control. These effects should be more pronounced in the absence of visual information across all three experiments.

## Experiment 1

### Methods

#### Subjects

Forty-four subjects (27 female and 17 male) aged from 19 to 61 years (*M* = 28.68, SD = 9.88) voluntarily participated in the experiment. The subjects, mostly students, were recruited by approaching them as they walked across the campus of the University of Mainz. All of them followed instructions of the experiment and were included in the data analysis. Informed consent was obtained beforehand verbally from all subjects, and they were debriefed after the experiment.

#### Design

The study was a field experiment with a multifactorial within-subjects-design to investigate the influence of a difficult balancing task on time perception. The subjects had to perform a temporal production task for intervals of either 10 or 15 s (time interval), with their eyes either open or closed (visual condition), and while standing either on one foot or on both feet (balance condition). These three factors were fully crossed. The visual condition was blocked, that is either all trials with open eyes or all trials with closed eyes were performed first. Within each visual condition, subjects first completed the two trials of the one balance condition, then those of the other. Per subject, the order of the balance conditions was held constant in both visual conditions. Likewise, the order of the two- time intervals was set constant for a given subject and counterbalanced among subjects, such that one-half of the subjects started each combination of visual condition and balance condition with the 10-s interval, and the other half with the 15-s interval. Each subject completed eight trials, one for each combination of visual condition, balance condition, and time interval.

#### Procedure

The data collection took place during 3 days in the period starting from September 14 to 20, 2021. All subjects were approached individually on campus. After being instructed that they would perform a time estimation task eight times while having their eyes open or closed, and while standing on one foot or on both feet, they gave their verbal consent. For the one-foot stand, they were instructed to lift the leg of their choice so that the foot was clearly off the ground. The length of the time interval to be produced was communicated verbally. Once this was done, after a few seconds, the experimenter gave the start signal, the German equivalent of “Ready-steady-go!” (“Auf die Plätze, fertig, los!”), at which time the stopwatch was started on a smartphone (iPhone). Subjects were asked to say “stop” out loud when they thought the predefined time interval had elapsed. For each trial, the experimenter recorded the duration of the produced time interval as indicated by the stopwatch. After completion of the last trial, demographic information and ratings concerning task difficulty experienced when balancing on one foot were obtained. Finally, the subjects were debriefed about the nature and the purpose of the experiment.

### Results and Discussion

We analyzed the produced time intervals in terms of the relative estimation error, which is given by


$$\frac{\mathrm{int}erval_{\text{produced}}-\mathrm{int}erval_{\text{to-be-produced}}}{\mathrm{int}erval_{\text{to-be-produced}}\;}\;\cdot 100\;[\%]$$

where *interval*_produced_ is the duration of the produced time interval, and *interval*_to-be-produced_ is the actual duration of the to-be-produced time interval in units of seconds, respectively. The resulting unit is %.

**Figure 2:**
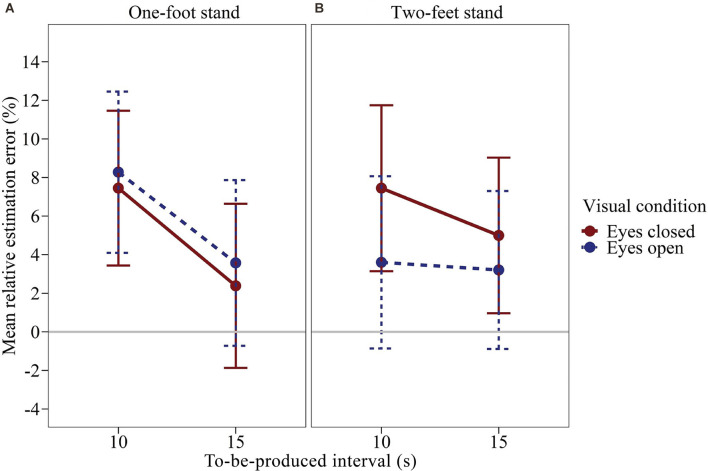
Mean relative estimation error of to-be-produced intervals by visual condition for each balance condition of Experiment 1. **(A)** One-foot stand condition, **(B)** two-feet stand condition. Error bars represent ±1 standard error of the mean (SEM). Values above 0 indicate relative overproduction of the interval.

We calculated a time interval × visual condition × balance condition repeated-measures ANOVA on the relative estimation error using a univariate approach. Figure 2 shows the mean relative estimation error as a function of the to-be-judged time interval, visual condition, and balance condition. The effects of visual condition and balance condition were clearly not significant, *F*_(1,43)_ = 0.396, *p* = 0.533, *η*^2^_p_ = 0.009 and *F*_(1,43)_ = 0.139, *p* = 0.711, *η*^2^_p_ = 0.003. The visual condition × balance condition interaction was not significant, *F*_(1,43)_ = 3.044, *p* = 0.088, *η*^2^_p_ = 0.066. However, an interesting trend can be observed in the data. As can be seen in Figure 2; there is a trend for the visual condition to have an effect during the two-feet stand but not the one-foot stand. When standing on both feet, subjects produced longer durations with their eyes closed compared to open, *Δ*_mean_ = 2.82%, *SE*_Δ_ = 1.75%, Cohen’s (1988) *d*_z_ = 0.242. When standing on one foot, the effect of the visual condition was considerably attenuated and opposite, *Δ*_mean_ = −1.00%, *SE*_Δ_ = 1.87%, *d*_z_ = −0.081. The effect of the time interval was significant, *F*_(1,43)_ = 5.182, *p* = 0.028, *η*^2^_p_ = 0.108. On average, the relative estimation error was larger for the 10 s interval than for the 15 s interval. Descriptively, the effect of the time interval was more pronounced when standing on one foot. However, in the rmANOVA the time interval × balance condition interaction was not significant, *F*_(1,43)_ = 2.897, *p* = 0.096, *η*^2^_p_ = 0.063. Neither were the remaining effects, *F* ≤ 0.431, *p* ≥ 0.515. Across all conditions, subjects slightly overproduced the given time intervals (*M* = 5.12%, *SM*_M_ = 3.86%).

Our results show a trend towards longer interval productions with eyes closed compared to open when subjects were standing on both feet. In the one-foot condition, the visual condition had virtually no effect. We assume the following aspects to be responsible for this inconclusive pattern of results. First, the high strain in the one-foot condition may have made the estimates in both conditions less precise. This is more of a challenge than one may think. Many of our subjects had great difficulty balancing on one foot for the required durations, in particular with their eyes closed. Most subjects had to use their second foot briefly in between to keep their balance. Note that based on the trial sequences used in Experiment 1, we cannot perform an analysis devoid of a potential carry-over effect of the one-foot stand on the two-feet condition. Additionally, a physically demanding task such as this might have increased the level of the physiological arousal that is known to affect time perception (Droit-Volet et al., 2004; Grommet et al., 2011; Kroger-Costa et al., 2013; Rey et al., 2017). Second, our subjects performed only two trials per combination of visual condition and balance condition. Thus, also for the two-feet stand, for which we found a quite promising trend, we could only measure the effect of the visual condition rather coarsely. For these reasons, we conducted a second experiment in which we focused on the two-feet stand and collected twice the number of interval productions per visual condition.

## Experiment 2

### Methods

#### Subjects

Forty-eight subjects (22 female, 26 male) aged from 17 to 63 years (*M* = 26.13, SD = 8.99) participated in the experiment. The recruitment procedure was the same as in Experiment 1. None of the subjects had participated in Experiment 1 and none had to be excluded from the data analysis. The experimenter obtained verbal informed consent beforehand and debriefed the subjects after the experiment.

#### Design and Procedure

The data collection took place during 3 days in the period from October 1 to October 7, 2021. The design of the experiment was similar to Experiment 1 and investigated the influence of balancing on time perception using an easier balancing task. Subjects were instructed to perform temporal productions of intervals of either 5, 10, 15, and 20 s (time interval) with their eyes open and closed (visual condition) while standing on both feet. The visual condition was blocked. One half of the subjects first completed all trials with their eyes open and then the other half with their eyes closed. The order of the four intervals to be judged was the same for the two blocks for a given subject but was counterbalanced between subjects, so that two subjects were assigned to each of the 24 possible orders. In total, each subject completed eight trials. In all other respects, the procedure was identical to that of Experiment 1.

### Results and Discussion

**Figure 3:**
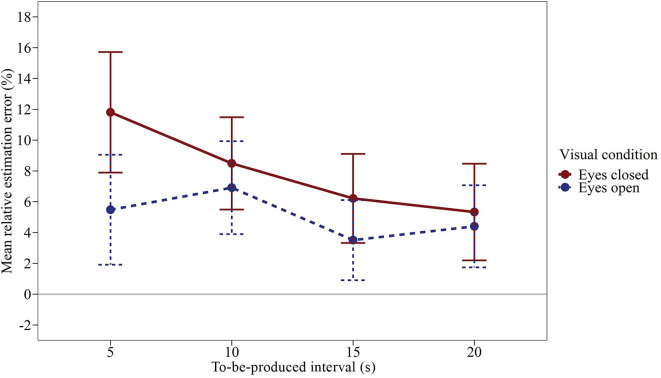
Mean relative estimation error of to-be-produced intervals by visual condition of Experiment 2. Error bars represent ±1 standard error of the mean (SEM). Values above 0 indicate relative overproduction of the interval.

We ran a time interval × visual condition rmANOVA using a univariate approach with Huynh and Feldt (1976) correction for the degrees of freedom (correction factor ε). Figure 3 shows the mean relative estimation errors (calculated as in Experiment 1) as a function of the duration of the to-be-produced time interval and the visual condition. The effect of the visual condition was significant, *F*_(1,47)_ = 5.719, *p* = 0.021, *η*^2^_p_ = 0.108. Subjects produced longer time intervals with their eyes closed compared to open eyes, *Δ*_mean_ = 2.88%, *SE*_Δ_ = 1.21%, *d*_z_ = 0.345. The effect of the time interval was not significant, *F*_(3,141)_ = 2.503, *p* = 0.089, *η*^2^_p_ = 0.051, ε = 0.642, accompanied by a significant visual condition × time interval interaction, *F*_(3,141)_ = 2.717, *p* = 0.047, *η*^2^_p_ = 0.055, ε = 1.00. As illustrated in Figure 3; the mean relative estimation error slightly decreased with increasing interval duration, especially in the interval productions with closed eyes. As in Experiment 1, averaged across all conditions, subjects slightly overproduced the given time intervals (*M* = 6.52%, *SM*_M_ = 2.81%).

Taken together, the results of Experiments 1 and 2 show that in an easy active balancing task, i.e., standing still on both feet, subjects with their eyes closed produced longer intervals compared to the eyes open condition. How can this effect be explained? The active balancing task, which we perform casually and without notice in everyday life, becomes somewhat of a challenge when we close our eyes. The increased effort that is involved in the motor control, as well as the increased postural sway that goes along with balancing in the dark (Era et al., 2006; Hansson et al., 2010), and the involvement of the vestibular afferent information could be responsible. Note, however, that the overproduction is opposite to the above-mentioned finding that time productions in microgravity are underproduced (Semjen et al., 1998). To remove the potential effects of physical effort and attention to the motor control task from the equation, we decided to forego active balancing in a third experiment. We placed subjects on a swing to isolate the potential effect of passive vestibular stimulation on time perception.

## Experiment 3

### Methods

#### Subjects

Forty-eight subjects (30 female, 18 male) aged from 18 to 58 years (*M* = 28.73, SD = 9.85) participated in the experiment. They were recruited by approaching them at a public playground, near a student accommodation, or as they were walking by. None of the subjects had to be excluded from the data analysis. As before, informed consent was obtained verbally, and they were debriefed after the experiment.

#### Design

In Experiment 3, we investigated the influence of the passive vestibular stimulation on the interval production task used in Experiments 1 and 2. The design was identical to that of Experiment 2, with the exception that instead of the active balancing task, vestibular stimulation was induced purely passively. Subjects were instructed to relax comfortably into a large swing, which was brought into motion by the experimenter (Figure 1). The swing chosen for the experiment was a playground nest swing that could comfortably accommodate an adult person. The subject assumed a lying posture, such that the main body axis was aligned with the swing plane. The distance of the nest from the ground was 41 cm, and it was suspended at a radial distance of 178.5 cm from the fulcrum on a supporting beam. For the vestibular stimulation, upon embarkation of the subject, the experimenter moved the swing to a starting position of 18° from the resting position, such that a full oscillation cycle spanned an amplitude of 36° and took 2.48 s to complete. The pushes were always given from behind (i.e., outside the subject’s field of view) in the direction of oscillation. Subjects performed the same temporal production task of intervals of either 5, 10, 15, or 20 s (time interval) with their eyes either open or closed (visual condition), as in Experiment 2. They were instructed to relax and lie still in the nest of the swing. The experimenter could either hold the swing still or swing it (vestibular stimulation). These three factors were fully crossed. The swing motion was blocked, that is either all trials lying still in the nest or all trials with the movement were performed first. Within each of these blocks, one half of the subjects first completed all trials with their eyes open and the other half with their eyes closed. For the combinations of vestibular stimulation and visual condition, the order of the four intervals to be judged was set constant within a given subject and counterbalanced among subjects. Apart from that, the procedure was identical to that of Experiment 2. In total, Experiment 3 consisted of 16 trials.

#### Procedure

The data collection took place for 5 days between October 25 and November 6, 2021. As before, the length of the time interval to be produced was communicated verbally, as were the experimenter’s start and the subject’s stop signals. Regardless of the swing motion condition, subjects had to maintain the same relaxed position for the entire experiment, which lasted approximately 10 min. During the movement of the swing, subjects were only given slight booster pushes between the single trials to maintain the 36°- amplitude. Thus, the pushing did not interfere with the time estimation. For each trial, a second experimenter recorded the produced time as stopped with the stopwatch.

### Results and Discussion

**Figure 4:**
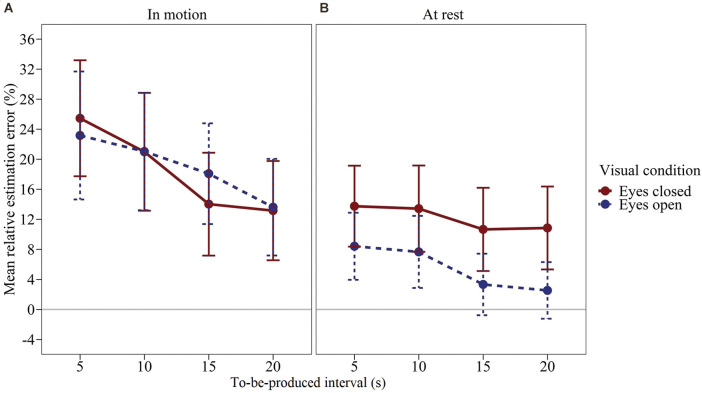
Mean relative estimation error of to-be-produced intervals by visual condition for each vestibular stimulation condition of Experiment 3. **(A)** Swing in motion, **(B)** Swing at rest. Error bars represent ±1 standard error of the mean (SEM). Values above 0 indicate relative overproduction of the interval.

We computed a time interval × visual condition × vestibular stimulation rmANOVA using the same specifications as in Experiment 2.The effect of vestibular stimulation was significant, *F*_(1,47)_ = 7.818, *p* = 0.007, *η*^2^_p_ = 0.143. Figure 4 shows the mean relative estimation error for the produced time intervals (calculated as before) as a function of the time interval, visual condition, and vestibular stimulation. Averaged across all combinations of visual conditions and time intervals, subjects produced longer intervals when the swing was in motion (see Figure 4), *Δ*_mean_ = 9.86%, *SE*_Δ_ = 3.53%, *d*_z_ = 0.404. The effect of the visual condition was not significant, *F*_(1,47)_ = 2.605, *p* = 0.113, *η*^2^_p_ = 0.053. However, there was a significant visual condition × vestibular stimulation interaction, *F*_(1,47)_ = 4.809, *p* = 0.033, *η*^2^_p_ = 0.093. To investigate this interaction in more detail, we compared the mean estimation error for eyes closed vs. eyes open separately for each of the two levels of vestibular stimulation by means of a paired-samples t-test (two-tailed). When the swing was at rest, the effect of visual condition was significant, *t*_(47)_ = 2.365, *p* = 0.022. In contrast, when the swing was in motion, the effect of visual condition was clearly not significant, *t*_(47)_ = −0.262, *p* = 0.795. As illustrated in Figure 4; when the swing was at rest, subjects produced longer time intervals with their eyes closed compared to open, *Δ*_mean_ = 6.69%, *SE*_Δ_ = 2.83%, *d*_z_ = 0.341. In contrast, when the swing was in motion, the interval productions were largely unaffected by the visual condition, *Δ*_mean_ = −0.57%, *SE*_Δ_ = 2.16%, *d*_z_ = −0.038. In the rmANOVA, all remaining effects were not significant, *F* ≤ 1.562, *p* ≥ 0.213. Across all conditions, subjects overproduced the given time intervals more clearly than in Experiments 1 and 2 (*M* = 13.76%, *SE*_M_ = 5.56%).

In sum, the results of Experiment 3 show that passive vestibular stimulation equally leads to longer interval productions. In addition, as in Experiment 1 (although not significantly there), we found that otherwise vestibular stimulation modulated the effect of the visual condition. When the nest swing was at rest, subjects produced longer time intervals when their eyes were closed compared to open. When, however, the swing was in motion, the effect of the visual condition was eliminated completely.

## General Discussion

We have conducted three experiments to investigate the effect of vestibular stimulation on temporal productions of supra-second time intervals. It is exceedingly difficult, if not impossible, to isolate vestibular influence, and the time range of the stimuli is often critical. Often, the vestibular stimulation is potentially confounded by other factors such as tactile and postural stimulation, physical and/or physiological stress, memory effects, or the choice of the motor actions that are used to signal a temporal judgment. Previous research on this topic has remained inconclusive, and the number of studies that have used supra-second intervals is rather limited. In order to examine the influence of the vestibular system on time perception in the supra-second range, we started with a most demanding active balancing task in Experiment 1 and then made the task easier in Experiment 2, and finally provided mere passive vestibular stimulation in Experiment 3. We have covered the range from balancing on one foot with eyes closed to being gently rocked back and forth by the oscillatory movement of a swing. To minimize the potential effects of memory and motor response as far as possible, we employed a task in which subjects verbally produced predetermined supra-second time intervals in the range of 5–20 s duration. By and large, vestibular stimulation caused an overproduction of the time intervals.

Let us now take a closer look at the SET model outlined in the introduction. An over-production can be attributed to a slowing of the pacemaker or to the accumulator missing some of the pulses produced by an unchanged pacemaker. In Experiment 1, participants had to perform the temporal productions while engaged in an active balancing task requiring tactile and vestibular motor control, with their eyes closed or open. This task was exceedingly difficult for most of our subjects when their eyes were closed. Thus, it seems safe to assume that the strain of the balancing task should have stimulated the pacemaker and thus increased the rate at which it generated pulses. Accordingly, one would have expected shorter temporal productions in the one-foot condition compared to two-feet condition as well as with eyes closed compared to open. However, this was not the case. Note that the expected shorter interval productions and the associated accelerated passage of subjective time presuppose an unchanged accumulator. More pulses are emitted (and properly accumulated) per physical unit of time, which should lead to an overestimation of time passed and, thus, to shorter productions. Yet again, we found no such effect of the balancing task. Nor did the availability of visual input matter, maybe with the exception of a tendency for an interaction between balance and visual condition. When standing on both feet, participants tended to overproduce temporal intervals with eyes closed compared to eyes open. This runs opposite to the expected increase in pacemaker arousal. Thus, if one were to interpret this trend, one would have to attribute it to attention on the part of the accumulator. However, if balancing strain would have caused the accumulator to miss pulses, there should have been an effect of vision in particular in the one-foot condition. This was not the case.

Considering that the one-foot balancing task in Experiment 1 was overly difficult and because of its difficulty surely has introduced a lot of postural sway (Era et al., 2006; Hansson et al., 2010), we sought to reduce variability in Experiment 2. Subjects stood on both legs while making temporal productions over a wider range of time intervals with eyes closed or open. Here, once again, subjects overproduced the instructed durations, but they did more strongly so when their eyes were closed, as compared to open. In other words, the everyday task of seemingly trivial active balancing, which is involved in standing upright with eyes closed, led to a contraction of subjective time. How can this finding be explained? Within the framework of the SET model, our results would be compatible with both a reduced pacemaker rate and a reduced recording of pulses by the accumulator. In the following, we will elaborate on why we consider a reduced recording by the accumulator to be the most likely explanation. To start on the side of the pacemaker, higher physiological arousal due to the more demanding balancing task in the condition with eyes closed may have influenced the rate of the pacemaker. However, based on the results of Experiment 1, we deem the level of physiological arousal unlikely to account for the pattern of results. Moreover, when considering upright posture maintenance with eyes closed as a more arousal-inducing physical activity than standing upright with eyes open, one would expect an acceleration in the pulse rate of the pacemaker when the eyes are closed, which in turn should lead to shorter rather than longer interval productions. In line with this conclusion, physical stress, such as through induced muscle tension (Warm et al., 1967), pedaling on a cycle ergometer (Vercruyssen et al., 1989), or running on a treadmill (Kroger-Costa et al., 2013), all are associated with dilation of subjective time, that is an overestimation of temporal durations. If physical stress or arousal of this kind were at the heart of our balancing tasks, we should have found underproduction rather than overproduction of the instructed intervals. Thus, our results cannot be explained by pacemaker arousal.

In contrast, on the side of the accumulator, our results could be attributed to the diversion of attentional resources (Brown, 1997) or a comparable inefficiency in the way the pacemaker pulses are counted. When performing the timing task while balancing, attentional resources might be allocated between the timing and secondary non-timing tasks. As more resources are dedicated to the secondary task, fewer resources are available for timing, which leads the accumulator to miss pulses emitted by the pacemaker (Zakay and Block, 1996). Is this a likely explanation? At first sight, this does seem so. For instance, it has been reported that subjects overestimated durations following interoceptive mindfulness meditation (Kramer et al., 2013), which focuses attention. In the same vein, interoceptive awareness and attention to one’s own heartbeat were associated with longer produced time intervals in the range of 8–20 s (Meissner and Wittmann, 2011). As already mentioned in the introduction, participants tended to underestimate the durations of events when engaged in secondary tasks requiring a greater amount of attentional and cognitive resources (Thomas and Weaver, 1975; Zakay and Block, 1996; Brown and Boltz, 2002). Balancing with eyes closed can be regarded as a secondary task that diverted attention away from the accumulator, which could then have caused temporal overproduction. In other words, the criterion of the instructed duration was reached later as the accumulation of the arriving pulses built up more slowly because some pulses were missed by the accumulator. Thus, reduced attention caused by the balancing task is compatible with the findings of Experiment 2. Note, however, that in Experiment 1, the exceedingly difficult one-foot balancing task should have demanded the most attentional resources but did not produce longer time estimates than the much easier two-feet balancing task.

Could the effects be attributed to the engagement of the motor system with or without negligible vestibular contribution? In a recent study, Castellotti et al. (2022) found that a secondary cognitive task led to an underestimation of a given time interval between 15 and 120 s. This could be an attention effect. Walking on a treadmill, as opposed to sitting while solving arithmetic tasks, led to even more pronounced underestimation. When assuming that no further attention was needed to walk on the treadmill, this could be a mere motor effect. This is consistent with our results, but note that together with the results of our third experiment, it is unlikely that the mere motor exertion is responsible here. Lying in the swing required neither attention nor motor exertion. Thus, vestibular stimulation appears to have been critical in Castellotti et al.’s walking condition.

Could mere vestibular stimulation have slowed the pacemaker or distracted the accumulator? To further investigate the role of vestibular load in timing, Experiment 3 examined the effects of vestibular stimulation through passive oscillatory movements induced by a swing. Here, participants produced longer intervals during pronounced passive vestibular stimulation compared to when the swing was at rest. Interestingly, eye closure had no effect when the swing was in motion. In contrast, when the swing was at rest, participants produced longer temporal intervals with their eyes closed compared to open, which replicates the findings of Experiment 2. Also, the effect of the vestibular stimulation was larger than that of eye closure at rest. Taken together, this does indeed suggest a direct vestibular effect on the timing network. Within the SET framework, and given that active balancing with eyes closed is strenuous and should—if anything—speed up the pacemaker, the vestibular effect suggests that the accumulator has missed pulses rather than the pacemaker having produced fewer pulses. Accumulator misses are the most likely explanation for the overproduction of temporal intervals under the conditions of vestibular stimulation.

Given the existing research on the vestibular system and the cerebellum, we could make tentative claims regarding the brain regions involved in the temporal processing of supra-second intervals. When considering the crucial role of the cerebellum in the functions of vestibular control and proprioception, the results of Experiments 2 and 3 are in line with the findings of Gooch et al. (2010), who found that cerebellar damage led to the contraction of subjective time for supra-second intervals. However, cerebellar involvement in this range needs to be replicated, which so far has only been established for sub-second intervals (Schubotz et al., 2000; Koch et al., 2007). That said, it could be argued that the tasks used in our experiments do not only affect cerebellar activation but also other brain regions further downstream, such as the basal ganglia. The basal ganglia have also been implicated in vestibular and motor control functions (Stiles and Smith, 2015). An fMRI study reported the activation of the basal ganglia in duration discrimination tasks (Rao et al., 2001). Pathology of these brain regions in patients with Parkinson’s disease has likewise been linked to overestimation and underproduction of supra-second time intervals (Pastor et al., 1992). However, other brain regions are involved as well, for instance, dorsolateral pre-frontal cortex (Lewis and Miall, 2003) to which the basal ganglia have extended connections (Alexander et al., 1986). Further brain imaging and stimulation studies are needed before we can pin down the brain regions associated with vestibular timing tasks.

In sum, the results of Experiments 1–3 demonstrate the influence of vestibular stimulation on temporal processing. Note that the degree of over-production, up to 25% for the 5-s interval, was larger for the vestibular stimulation used in Experiment 3, as compared to the more or less strenuous balancing tasks used in Experiments 1 and 2. We can be reasonably certain, that memory encoding differences can be ruled out as explanations of our results. Time intervals that were encoded at rest and reproduced during the stress of running on a treadmill were under-produced, as is compatible with an accelerated pacemaker during exercise (Sayalı et al., 2018). By using pre-defined interval lengths and by the absence of balancing effects, memory encoding cannot explain our results. Neither can arousal on the side of the pacemaker. Mere stimulation of the vestibular system, accompanied by those tactile and proprioceptive cues that are necessarily confounded with it, distorted time perception in the direction of subjective temporal contraction. Vestibular stimulation prompted our subjects to produce lengthened time intervals, which can be interpreted as an impact on the accumulator that lets it miss pulses. Thus, vestibular activation can be said to perceptually shorten a given time interval.

It is important to note, however, that the methods employed in the current experiments were rather crude. We used a stopwatch in a field setting, which forced us to focus on long time intervals of up to 20 s and may have introduced a degree of inaccuracy, especially for the shortest intervals. Despite this limitation, the effects on temporal performance that we did find in the field, provide strong support for the involvement of the vestibular system in timing functions. Future studies in a controlled laboratory setting should extend this finding to shorter intervals and control for arousal to further specify the involvement of the vestibular system in temporal processing. Furthermore, taking into account the inconsistent findings of the previous studies regarding the role of the vestibular system and the cerebellum, in particular, brain imaging and stimulation studies are crucial for further research on their involvement in time perception. For instance, the application of peripheral electrical stimulation could be thought of as distracting noise in the system. Thus, we might predict that with galvanic stimulation, the effect of vestibular stimulation found here will disappear as the noise leads to a down-weighting of the vestibular signal. An opposite effect might arise from cerebellar stimulation. Additionally, by applying systematic classical galvanic stimulation to the mastoid regions (see e.g., Day, 1999), one could stimulate vestibular afferents to generate percepts that are similar to those generated by the movement of the swing in our third experiment. If we find comparable effects of time contraction with such galvanic stimulation in the stationary observer, that is in the absence of the dynamically changing proprioceptive information or changes in the positions of the body present during the oscillatory motions of the swing, the role of the vestibular system in time production could be isolated and pinpointed further. This line of research is worth future investigation since it has important implications for tasks and activities that require spatial navigation and orientation, where accurate temporal estimation is essential, such as during the operation of a vehicle or an aircraft.

On a final note, if the effect of time contraction is not a maladaptive side-effect but rather has adaptive functionality, what could it be? During rapid body accelerations, the body is typically at higher risk to bump into things and in more immediate need to initiate swift action or corrective posture changes. The subjective time contraction could bear witness of a sharpened perceptual state which is induced by the vestibular stimulation. Be this as it may, the interaction between the vestibular system and time perception opens up a few interesting questions for future research.

## Data Availability Statement

The raw data of Experiments 1–3 can be retrieved from: https://osf.io/z7fg2/?view_only=1b665a41ca8144268e1b48ed0832eeca.

## Ethics Statement

Ethical review and approval was not required for the study on human participants in accordance with the local legislation and institutional requirements. Written informed consent for participation was not required for this study in accordance with the national legislation and the institutional requirements.

## Author Contributions

All authors were involved in designing the experiments. CC processed the data and performed the analysis. All authors interpreted the data and discussed the results. NU and CC wrote the first draft of the manuscript. All authors contributed to the article and approved the submitted version.

## Conflict of Interest

The authors declare that the research was conducted in the absence of any commercial or financial relationships that could be construed as a potential conflict of interest.

## Publisher’s Note

All claims expressed in this article are solely those of the authors and do not necessarily represent those of their affiliated organizations, or those of the publisher, the editors and the reviewers. Any product that may be evaluated in this article, or claim that may be made by its manufacturer, is not guaranteed or endorsed by the publisher.
